# Exosomes from acellular Wharton’s jelly of the human umbilical cord promotes skin wound healing

**DOI:** 10.1186/s13287-018-0921-2

**Published:** 2018-07-13

**Authors:** Nazihah Bakhtyar, Marc G. Jeschke, Elaine Herer, Mohammadali Sheikholeslam, Saeid Amini-Nik

**Affiliations:** 1 Sunnybrook Research Institute, Sunnybrook’s Trauma, Emergency & Critical Care (TECC) Program, Ross Tilley Burn Centre, Office: M7-161, Lab: M7-140, 2075 Bayview Ave., Toronto, ON M4N 3M5 Canada; 2 The University of Toronto, Institute of Medical Science, Toronto, ON Canada; 3 Division of Plastic and Reconstructive Surgery, Department of Surgery, The University of Toronto, Toronto, ON Canada; 4 Department of Laboratory Medicine and Pathobiology (LMP), The University of Toronto, Toronto, ON Canada; 5 Gynecology and Obstetrics Department, Sunnybrook Health Sciences Centre, Toronto, ON Canada

**Keywords:** Skin, Umbilical cord, Wound healing, Wharton’s jelly, Exosomes, Stem cells

## Abstract

**Background:**

Compromised wound healing has become a global public health challenge which presents a significant psychological, financial, and emotional burden on patients and physicians. We recently reported that acellular gelatinous Wharton’s jelly of the human umbilical cord enhances skin wound healing in vitro and in vivo in a murine model; however, the key player in the jelly which enhances wound healing is still unknown.

**Methods:**

We performed mass spectrometry on acellular gelatinous Wharton’s jelly to elucidate the chemical structures of the molecules. Using an ultracentrifugation protocol, we isolated exosomes and treated fibroblasts with these exosomes to assess their proliferation and migration. Mice were subjected to a full-thickness skin biopsy experiment and treated with either control vehicle or vehicle containing exosomes. Isolated exosomes were subjected to further mass spectrometry analysis to determine their cargo.

**Results:**

Subjecting the acellular gelatinous Wharton’s jelly to proteomics approaches, we detected a large amount of proteins that are characteristic of exosomes. Here, we show that the exosomes isolated from the acellular gelatinous Wharton’s jelly enhance cell viability and cell migration in vitro and enhance skin wound healing in the punch biopsy wound model in mice. Mass spectrometry analysis revealed that exosomes of Wharton’s jelly umbilical cord contain a large amount of alpha-2-macroglobulin, a protein which mimics the effect of acellular gelatinous Wharton’s jelly exosomes on wound healing.

**Conclusions:**

Exosomes are being enriched in the native niche of the umbilical cord and can enhance wound healing in vivo through their cargo. Exosomes from the acellular gelatinous Wharton’s jelly and the cargo protein alpha-2-macroglobulin have tremendous potential as a noncellular, off-the-shelf therapeutic modality for wound healing.

**Electronic supplementary material:**

The online version of this article (10.1186/s13287-018-0921-2) contains supplementary material, which is available to authorized users.

## Background

One of the most important functions of the skin is to be a barrier against the environment [1]. Insults such as burns, chronic skin ulcers as a result of pressure, venous stasis, or diabetes mellitus represent some of the conditions in which the tissue integrity is disrupted and a wound is created [1, 2]. According to the World Health Organization (WHO), burns are a global problem which account for approximately 180,000 deaths per year and, in 2004, nearly 11 million people around the world were burned severely enough to require medical care [3]. The high mortality in burn patients results from the loss of skin which increases metabolic demand, fluid loss, and enhances the risk of infection. Therefore, wound closure is imperative [4]. Furthermore, approximately 1.5 billion people suffer from inadequate wound healing due to a combination of progressive aging and the lack of adequate healthcare [4–7]. Diabetes, for example, is another prevalent condition that can lead to severe wounds. Diabetes can result in diabetic ulcers due to prolonged inflammation, a lack of neovascularization, reduced collagen production, high levels of proteinases and synthesis of collagen, and malfunctioning macrophages [8–11]. Therefore, to achieve the therapeutic goal of early and complete wound closure, wound healing research is critical.

Skin wound healing is a dynamic process that occurs after the tissue is damaged. Wound healing incorporates four phases which overlap: hemostasis, inflammation, tissue formation, and tissue remodeling [12, 13]. If the wound healing steps do not occur in a coordinated and timely manner, abnormal wound healing can result, and an open wound can lead to infection and inadequate thermal and fluid management. Autologous skin grafts are the first line of care. However, for wound closure, because native skin is limited, skin substitutes have been developed that have many benefits but also disadvantages in skin wound healing. The three most important components of a skin substitute are scaffolds, growth factors, and cells [4, 14–19]. Stem cell therapies have received much attention during the last decade, particularly for the management of deficient skin healing. One of the sources of stem cells used for wound healing is the umbilical cord both for deficient healing and, it has been suggested, as a possible remedy for excessive healing [20, 21]. The umbilical cord is therefore a highly promising area of wound healing research.

The umbilical cord contains two arteries and one vein which are enveloped by mucous connective tissue called Wharton’s jelly (WJ) [22]. In the Wharton’s jelly, the glycosaminoglycan hyaluronic acid is highly prevalent and forms a gel around fibroblasts and collagen fibrils which protects the tissue from pressure and maintains tissue architecture [23, 24]. Mesenchymal stromal cells (MSCs) can be found within various regions of the umbilical cord, such as the umbilical cord blood, the umbilical vein subendothelium, and Wharton’s jelly [25]. In 1991, McElreavey and his group isolated MSCs from the Wharton’s jelly [26]. Since then, many laboratories have identified MSC markers on cells from Wharton’s jelly and studied their properties as both embryonic and adult stem cells [27, 28]. Besides the cells itself, the paracrine factors secreted by MSCs have been reported to play a vital role in therapeutic, immunomodulatory, and tissue regeneration abilities of MSCs [29]. MSCs provide a supportive role and a microenvironment that allows for the culturing of hematopoietic stem cells (HSCs); it has been reported that MSCs secrete factors which enable the growth of HSCs ex vivo [30, 31]. We also reported that WJ-MSC conditioned medium with its secretory factors have positive effects on wound healing in vitro [21]. During wound healing, cell to cell communication is crucial [13, 32]. Multicellular organisms rely heavily on intercellular communication and this can be accomplished through both direct cell-cell contact and transfer of secreted molecules [32, 33]. In the last 20 years, another mode of intercellular communication has emerged; this is the intercellular transfer of extracellular vesicles (EVs), such as exosomes [32]. Exosomes are a form of extracellular vesicle. Exosomes are saucer-shaped vesicles between 30 and 100 nm in diameter; they are surrounded by a lipid bilayer and float at a density of 1.13–1.19 g/ml in sucrose gradients [34]. Vesicles with characteristics of exosomes have been isolated from multiple different body fluids, including semen, blood, saliva, breast milk, amniotic fluid, ascites fluid, cerebrospinal fluid, and bile [32, 35–38]. During the last decade, much research has focused on exosomes as a mode of intercellular communication since exosomes are secreted from various cell types including stem cells [39, 40]. There are reports that state that MSCs secrete exosomes in vitro and these exosomes have therapeutic benefits [41, 42]. The secretome within the umbilical cord Wharton’s jelly, much of which could be contributed by MSCs, is therefore a promising avenue of research.

Since the MSCs are embedded in a significant amount of extracellular matrix components within the umbilical cord, we recently asked if there are beneficial factors in the extracellular matrix devoid of cells which aid in skin wound healing. We reported that, indeed, acellular gelatinous Wharton’s jelly (AGWJ) has beneficial wound healing properties in vivo in a murine model by allowing for wound healing at an earlier time point concomitant with a significant reduction in wound length after AGWJ treatment. AGWJ also increased cell migration in vitro, and led to the expression of alpha-smooth muscle actin (αSMA), a marker of myofibroblasts [43]. Having investigated the effect of AGWJ on wound healing in vitro and in vivo, there were still many questions left unanswered with regards to the key ingredients within the AGWJ and the role these ingredients play in facilitating wound healing. In the present study, we further explored the mechanism of how AGWJ enhances wound healing. We show that exosomes isolated from AGWJ enhance cell viability and cell migration in vitro and enhance skin wound healing in the punch biopsy wound model in mice. These exosomes contain a large amount of alpha-2-macroglobulin (α2M), a protein that likely is the main component of exosomes in enhancing wound healing.

## Methods

### Cell culture

Skin dermal fibroblasts were isolated from healthy skin tissue samples. Briefly, dermal fibroblasts were isolated by first removing underlying fat from the dermis. The skin was then dissected into explant pieces of 2 to 4 mm and cultured in 10-cm dishes by placing the skin with the dermis side down on the plate. After allowing the skin to adhere to the plate (approximately 5 min), complete Dulbecco’s modified Eagle’s medium (DMEM) was added to the cell culture plate and placed in a 37 °C incubator. When fibroblast cells had migrated out of the tissue and onto the plate, the tissue pieces were removed. At a fibroblasts confluency of 70%, the cells were trypsinized with 0.05% trypsin in preparation for subculture. Fibroblasts were then subcultured in 75-cm^2^ tissue culture flasks at a density of 5000 cells/cm^2^. Tissue culture plastic ware was purchased from BD Falcon™ (Bedford, MA, USA), and all tissue/cell culture media and supplements were purchased from Wisent Inc. (St-Jean-Baptiste, QC, Canada) unless stated otherwise. The fibroblast cell culture medium used was high-glucose DMEM supplemented with 10% fetal bovine serum (FBS) and 1% antibiotic-antimycotic solution.

### Acellularization of Wharton’s jelly

Consent was obtained from patients after receiving ethical clearance. Cesarean sections were performed by surgeons in the Department of Gynecology and Obstetrics at Sunnybrook Health Sciences Centre to obtain sterile umbilical cords. Each cord, which is approximately 20 cm in length, was cut in half to wash them before Wharton’s jelly isolation. The cord pieces were washed by dipping them in a 2% antibiotic/antimycotic phosphate-buffered saline (PBS) solution followed by dipping them in a 1% antibiotic/antimycotic PBS solution. Thereafter, the cords were dissected with a scalpel to open them lengthwise through the middle to reveal the Wharton’s jelly contents within the cord. Next, the jelly was scraped out of the umbilical cord. The jelly was transferred into a 50-ml conical tube with 20 ml of 1× DMEM complete medium and resuspended vigorously using a 5-ml pipette to dissolve the jelly into the medium. The resuspended jelly in the medium was then centrifuged at 1400 rpm for 10 min. The cells and cord debris were pelleted.

### Exosome isolation

Exosomes were isolated using a differential centrifugation protocol. Acellularized Wharton’s jelly in DMEM was centrifuged at 300 g for 10 min to pellet the cell debris and to clear the solution of leftover cells. This was followed by collection of the supernatant which was transferred to a new 15-ml conical tube. This tube was then centrifuged at 2500 g for 30 min to remove apoptotic bodies. After this, the supernatant was again collected and transferred to a new tube. This supernatant was then centrifuged at 13,000 g for 1 h to remove platelets and extracellular vesicles using Greiner 15-ml tubes. The supernatant was again collected and centrifuged at 100,000 g for 70 min at 4 °C. The supernatant was then discarded because the pellet now contained exosomes. The exosomes pellet was resuspended in 1× PBS and stored at −80 °C for future use.

### Cell migration study: scratch assay

Human dermal fibroblasts were seeded in six-well plates at a density of 20,000 cells/well. When the wells had reached 100% confluency, two scratches were made with a 1000-μl pipette tip. The medium was then aspirated and the cells were washed with PBS. The PBS was then aspirated. One well was immediately stained with crystal violet as a 0 h time point. This was followed by the addition of treatment medium containing exosomes in DMEM, recombinant α2M in DMEM at varying concentrations (0, 100, 1000 ng), or complete DMEM control media to the cells. After 24 h, the cells were stained with crystal violet. Images were taken under a microscope (Zeiss) with 4× magnification. Eight images were taken per scratch which represents a technical replicate of 8 and biological replicate of 3 for the fibroblast cells. A blinded individual conducted the cell quantification using ImageJ software (National Institutes of Health, Bethesda, MD, USA). The cells within the scratch area were counted as the cells in the scratch zone.

### Immunofluorescent tracking of exosomes

EYFP-expressing murine fibroblasts were seeded in an eight-well chamber slide and grown for 24 h in DMEM. After 24 h, the cells were treated with red fluorescently labeled exosomes (excitation 460 nm, emission 650 nm) using an exosome labeling kit (Exo-Glow; Systems BioSciences, Palo Alto, CA, USA). The Exo-Red exosome label is based on acridine orange chemistry and is a nucleic acid selective fluorescent cationic dye. It is cell-permeable and interacts noncovalently with RNA by electrostatic attraction and DNA by intercalation*.* Briefly, 50 μl of 10× Exo-Red was added to 500 μl resuspended exosomes in 1× PBS. This solution was then incubated at 37 °C for 10 min. Labeling of the exosomes was halted by adding 100 μl of the ExoQuick-TC reagent and subsequent placement on ice for 30 min. The sample was then centrifuged for 3 min at 14,000 rpm. The supernatant was removed and the exosome pellet was resuspended in 500 μl 1× PBS. The exosomes were tracked using time lapse imaging using automated confocal fluorescence microscopy (Opera Phenix™ high-content screening system; PerkinElmer).

### In-vivo wound healing model

Eight C57/black 6 mice (8 weeks old, male, body weight 25 to 30 g) were obtained from Jackson Laboratory under the guidelines of the Sunnybrook Research Institute and Sunnybrook Health Sciences Animal Policy and Welfare Committee of the University of Toronto. Animal procedures were reviewed and approved by Sunnybrook Research Institute and Sunnybrook Health Sciences Centre at the University of Toronto animal care and use committee. The animals were anesthetized and back cutaneous hair was removed by electrical shaving under anesthesia as stated in the Animal Protocol. Four 6-mm diameter full-thickness skin wounds were created on each side of the midline using an Integra Miltex disposable Biopsy Punch (Integra Life Sciences, Plainsboro, NJ, USA). The animals were randomly divided into two groups: treatment (exosomes and matrigel; BD Biosciences, San Jose, CA, USA) and control (complete DMEM and matrigel). Animals were monitored for 7 days before euthanasia and harvesting of wounds. Four mice received control treatment and four mice received exosome treatment. Each wound topically received 100 μl exosome treatment or control DMEM in matrigel mix. The treatment was added using a pipette with a P1000 tip. The day of the wounding was counted as day 0. On days 2 and 4 the wounds received a fresh application of the exosome treatment or the DMEM control. On day 6, 24 h before sacrificing the mice, the animals received an intraperitoneal injection of bromodeoxyuridine (BrdU; Calbiochem, San Diego, CA, USA).

### Murine skin histology

Wound skin from each group was subjected to fixation. Histological assessment was conducted on wound sections obtained from the widest part of the wound (the wound center). Hence, the most disrupted/least healed part of the wound was considered for healing analysis. This strategy allowed for the evaluation of the distinct changes in the wound healing process. Tissue sections were fixed in 10% buffered formalin at room temperature overnight. Next, the samples were transferred to 70% ethanol for another 48 h, and then embedded in paraffin. A serial section of the wound was performed at 5-μm cross-sections. The largest wound diameter or central wound section was stained for Masson’s trichrome staining.

### Masson’s trichrome staining

Paraffin-embedded slides were heated for 30 min at 60 °C. The slides were then deparaffinized with citrosol, followed by rehydration through 100% × 2, 95%, 70%, and washed in distilled water. Slides were placed in Bouin’s solution (26367–01; EMS, Hatfield, PA, USA) overnight at room temperature and washed. Hematoxylin stain (HHS16; Sigma, Saint Louis, MO, USA) and Biebrich scarlet-acid fuchsin solution were applied sequentially for 10 min. After each stain the slides were washed. Next, slides were differentiated in phosphomolybdic–tungstic acid for 15 min, and transferred to aniline blue for 5 min. All slides were washed in distilled water and then differentiated in 1% acetic acid for 2 min. Slides were dehydrated through 95% ethanol and absolute ethanol followed by clearing in citrosol. Slides were mounted with SHUR/Mount xylene-based liquid mounting media (Triangle Biomedical Sciences, Durham, NC, USA). Images were acquired using a Zeiss Axiovert 200 light microscope at 2.5×, 5×, and 40× magnification [4, 43].

### Western blot

Briefly, cell lysates and exosomal lysates (30 μg of protein per well) were separated by 10% SDS-PAGE gel and proteins were then transferred to nitrocellulose membrane, after which the blots were blocked with 5% skimmed milk in TBST buffer for 1 h. The blots were washed three times in TBST buffer for 10 min each and then blots were probed using the mouse monoclonal αSMA (anti-αSMA, 1:1000; clone 1A4; ebioscience, San Diego, CA, USA) and mouse monoclonal CD81 (anti-CD81, 1:100; Thermofisher Scientific, Waltham, MA, USA). Membranes were incubated with the primary antibody overnight at 4 °C and then detected the next day using their respective secondary antibodies. Bands were detected using the Biorad Chemidoc MP Imaging System.

### Mass spectrometry

Mass spectrometry (MS) was conducted at The SickKids Proteomics, Analytics, Robotics & Chemical Biology Centre (SPARC BioCentre) in Toronto, Ontario. Exosomes were first lysed using RIPA buffer to extract exosomal proteins. The samples were then delivered to SPARC. Samples were resuspended in 50 μl 50 mM NH_4_HCO_3_ (pH 8.3), and DTT was added to reduce cysteines at a final concentration of 10 mM. Cysteines were reduced at 60 °C for 1 h. The sample was cooled to room temperature, and iodoacetamide was added to a final volume of 20 mM. Samples were incubated at room temperature in the dark for 30 min. Iodoacetamide was then inactivated by adding DTT to a final concentration of 40 mM. MS-grade TPCK-treated trypsin (Promega) was added to a final protease:protein ratio of 1:50–1:100 and samples were digested overnight at 37 °C. The supernatant was removed from beads, lyophilized, and resuspended in 1% TFA. Peptides were purified by homemade C18 tips, and then lyophilized.

Samples were analyzed on an Orbitrap analyzer (Q-Exactive, ThermoFisher, San Jose, CA) outfitted with a nanospray source and EASY-nLC nano-LC system (ThermoFisher, San Jose, CA). Lyophilized peptide mixtures were dissolved in 0.1% formic acid and loaded onto a 75 μm × 50 cm PepMax RSLC EASY-Spray column filled with 2 μM C18 beads (ThermoFisher San, Jose CA) at a pressure of 800 Bar and a temperature of 60 °C. Peptides were eluted over 60 min at a rate of 250 nl/min using a gradient set up as 0–42% gradient of Buffer A (0.1% formic acid) and Buffer B (0.1% formic acid in 80% acetonitrile). Peptides were introduced by nano-electrospray into the Q-Exactive mass spectrometer (ThermoFisher, San Jose, CA). The instrument method consisted of one MS full scan (400–2000 m/z) in the Orbitrap mass analyzer with an automatic gain control (AGC) target of 1e6, maximum ion injection time of 120 ms and a resolution of 70,000 followed by 10 data-dependent MS/MS scans with a resolution of 17,500, an AGC target of 1e6, maximum ion time of 120 ms, and one microscan. Fragmentation occurred in the HCD trap with normalized collision energy set to 30. The dynamic exclusion was applied using a setting of 5 s.

### Transmission electron microscopy (TEM)

Briefly, exosomes were labeled with CD81 antibody and then treated with immunogold nanoparticles secondary antibody. Fibroblast cells were then treated with these labeled exosomes. After 2 hours the samples for TEM were fixed in 2% glutaraldehyde in 0.1 M sodium cacodylate buffer, rinsed in buffer, and fixed again in 1% osmium tetroxide in the buffer. Samples were then dehydrated in a graded ethanol series followed by propylene oxide and embedded in Quetol-Spurr resin. Sections (90 nm thick) were cut on a Leica EM UC7 ultramicrotome, stained with uranyl acetate and lead citrate, and viewed in an FEI Tecnai 20 TEM.

### Scanning electron microscopy (SEM)

Briefly, samples for SEM were fixed in 2% glutaraldehyde in cacodylate buffer, followed by rinsing in buffer and dehydration in a graded ethanol series. The samples were critical point dried in a Bal-tec CPD030 critical point dryer, mounted on aluminum stubs, gold coated in a Leica ACE200 sputter coater, and examined in an FEI XL30 SEM.

### Cell proliferation assay

Fibroblasts were seeded in an eight-well chamber slide at a concentration of 20,000 cells per well. After 24 h of cell growth, either DMEM, exosomes, or α2M were added at a concentration of 100 ng/ml α2M (R&D Systems, Oakville, ON; cat no. 1938-PI). After another 24 h, BrdU was added at a dilution of 1:200 and incubated for another 12 h and then analyzed using immunofluorescence microscopy. There were 3 biological replicates for this study and 3 technical replicates per experiment.

### Cell viability assay

Fibroblasts and keratinocytes were seeded in a 96-well clear-bottom tissue culture black plate (Corning Incorporated, Corning, NY; costar 3603) at a concentration of 10 cells/μl for a total of 1000 fibroblasts/well and 20 cells/μl for a total of 2000 keratinocytes/well. The fibroblasts were seeded in a separate 96-well plate to the keratinocytes. After 24 h, either DMEM (for fibroblasts) or Epilife medium (for keratinocytes), exosomes, or α2M was added at a concentration of 100 ng/ml (R&D Systems, Oakville, ON; cat. no. 1938-PI). After 24 h of incubation, Promega Glo luminescent reagent (Promega, Madison, WI; cat. no. G7571) was added to each well. The plate was shaken to completely mix the solution with the medium and then incubated for 10 min at room temperature. Cell viability was analyzed using luminescence and read using the Synergy H4 hybrid multimode microplate reader (100 Tigan Street Winooski, VT, USA). There were 3 biological replicates for the fibroblasts and 6 technical replicates per experiment. There was 1 biological sample of keratinocytes and 6 technical replicates.

### Analysis of collagen deposition in vivo

To determine the extent of collagen deposition in the granulation tissue of the in-vivo wound bed we used imageJ software to quantify collagen deposition from the Masson’s trichrome stained images. Each image was captured under 40× magnification by a blinded individual. Under the analyze function in imageJ, we used the Red Green Blue (RGB) histogram to determine the mean value for the blue color intensity, which in our trichrome images represent collagen. Furthermore, we measured the wound length. This was calculated using imageJ software to measure the length from one end of intact skin to the other end of intact skin which is surrounding the granulation tissue. Wound length was measured by a blinded individual.

### Statistical analysis

Experimental validity was confirmed by performing all in-vitro assays three times and the in-vivo experiment had four animals for each treatment group. All results are presented as mean ± standard deviation (SD) with 95% confidence interval after applying a Student’s *t* test using Microsoft Excel version 8. A *P* value ≤ 0.05 was considered as significant. Analysis of variance (ANOVA) was also used.

## Results

### AGWJ contains proteins characteristic of exosomes

We recently reported that AGWJ treatment of human fibroblasts and keratinocytes in vitro leads to enhanced cell migration of these cells, a key characteristic of skin wound healing [43]. Furthermore, we showed that AGWJ treatment enhances wound healing in vivo in mice [43]. Morphologically, the umbilical cord interior is highly composed of extracellular matrix as documented by SEM of a cross-section of the umbilical cord (Additional file 1: Figure S1). Since acellular Wharton’s jelly showed a beneficial effect on skin healing, and the extracellular matrix holds the Wharton’s jelly, we aimed to identify proteins that exist in AGWJ by performing mass spectrometry on umbilical cord AGWJ. Mass spectrometry results revealed isoform 2 of filamin-A to be highly expressed in AGWJ (Table 1), a protein which is enriched in exosomes [44, 45]. Using an ultracentrifugation protocol, we showed that, indeed, the acellular Wharton’s jelly of umbilical cords contains exosomes (Additional file 2: Figure S2).

**Table 1:** Mass spectrometry results of highly expressed proteins in acellular gelatinous Wharton’s jelly (AGWJ) from three separate umbilical cords

Abundant proteins in AGWJ	
Isoform 2 of Filamin-A OS=Homo sapiens GN=FLNA	
Isoform 2 of Myosin-11 OS=Homo sapiens GN = MYH11	
Actin, aortic smooth muscle OS=Homo sapiens GN = ACTA2 PE = 1 SV = 1	
Actin, alpha cardiac muscle 1 OS=Homo sapiens GN = ACTC1 PE = 1 SV = 1	
Actin, gamma-enteric smooth muscle OS=Homo sapiens GN = ACTG2 PE = 1 SV = 1	
Actin, cytoplasmic 1 OS=Homo sapiens GN = ACTB PE = 1 SV = 1	
Vimentin OS=Homo sapiens GN=VIM PE = 1 SV = 4	
Alpha-2-macroglobulin OS=Homo sapiens GN = A2M PE = 1 SV = 3	
Desmin OS=Homo sapiens GN = DES PE = 1 SV = 3	

*OS* Organism Name, *GN* Gene Name, *PE* Protein Existence, *SV* Sequence Version

### Exosomes isolated from Wharton’s jelly promote cell viability and cell migration in vitro

**Fig. 1:**
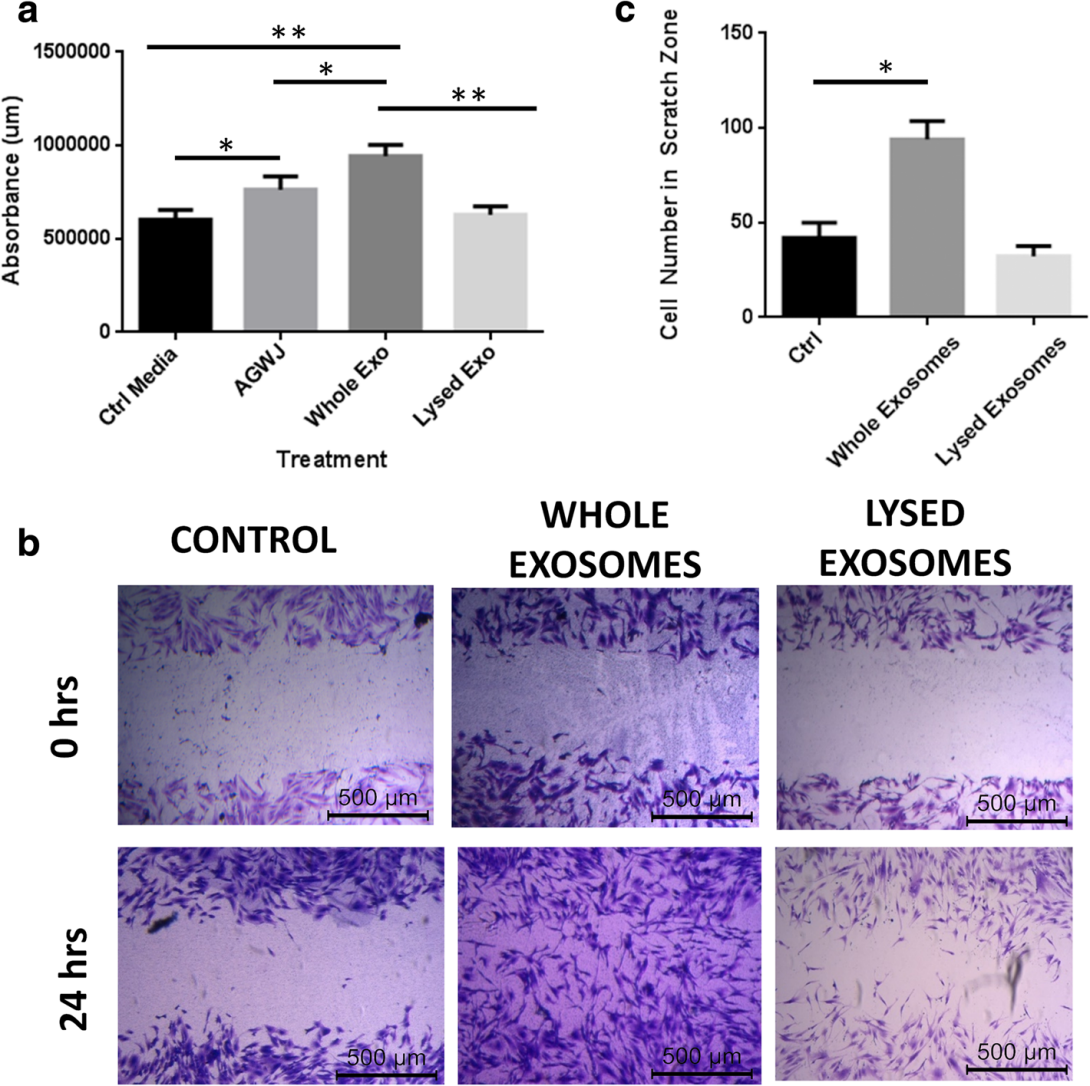
Analysis of whole exosome treatment on fibroblast viability and migration. **a** The absorbance level detected from the emitted luminescence from viable cells. Whole exosome (Exo)-treated fibroblasts had the highest viability, followed by acellular gelatinous Wharton’s jelly (AGWJ)-treated cells. Lysed exosomes and no treatment or control (Ctrl) medium-treated cells had the lowest viability. *N* = 3, *p < 0.05, **p < 0.01. **b** Crystal violet stained scratch assay showing fibroblast migration without any treatment, whole exosome-treated, and lysed exosome-treated cells at 0 h and 24 h. Cell migration was increased when the cells were treated with whole exosomes; however, lysed exosome-treated cells diminished cell migration after 24 h. **c** Quantification of the number of cells which have migrated into the scratch zone. Whole exosome-treated cells had a significantly increased migration compared with control medium-treated cells. *N* = 3; *p < 0.05

To verify whether the positive effect of AGWJ on skin healing is via the exosomes, we subjected fibroblasts to the AGWJ-derived exosomes and performed a viability assay as well as a scratch assay in vitro*.* For these assays, the exosomes were in two separate forms: the exosomes were either kept intact, surrounded by their membrane, or they were lysed open to reveal exosomal proteins. Viability was maximum when the cells were treated with whole exosomes in comparison with the other control treatments. It was significantly higher than AGWJ treatment, suggesting that exosomes are likely one of the main ingredients of the AGWJ in enhancing viability. A significant decrease in viability was observed in exosome-treated samples when the exosomes were lysed before treatment, suggesting that the content of the exosomes were diluted in the medium therefore diminishing its effect (Fig. 1a). Furthermore, cell migration was increased when a confluent monolayer of fibroblasts in culture was scratched, and the cells were treated with whole exosomes. On the other hand, when treated with lysed exosomes, the cells were less migratory compared with whole exosome-treated cells after 24 h (Fig. 1b, c). These data show that the exosomes in their intact form interact with cells and promote downstream effects and hence have an effect on wound healing properties such as cell viability and cell migration in vitro*.*

### Exosomes adhere to the fibroblast cell surface

**Fig. 2:**
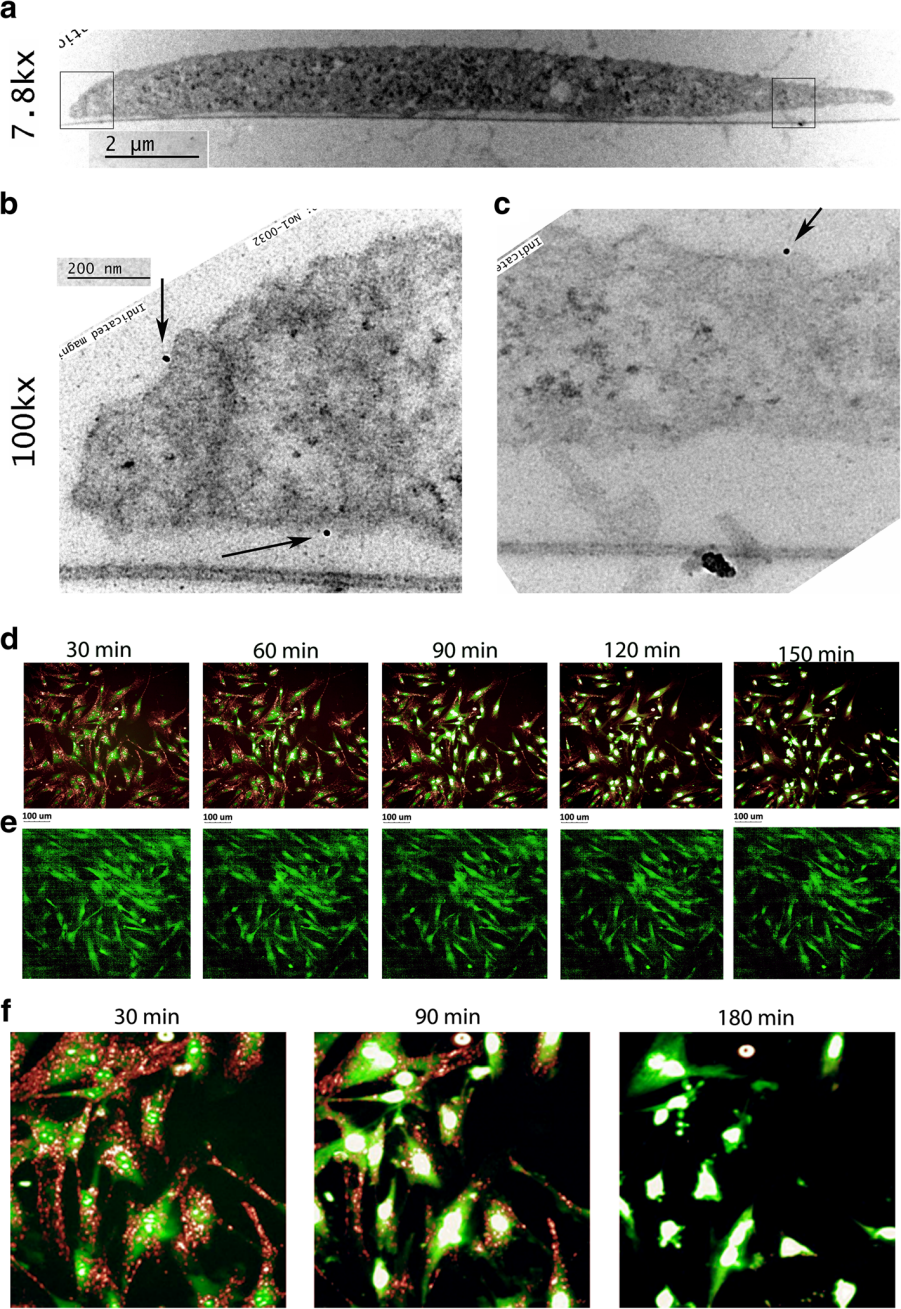
Analysis of exosome interaction with fibroblast cells Exosomes were labeled with CD81 antibody and treated with gold nanoparticles for visualization. **a** Transmission electron microscopy (TEM) at 7.8k× magnification showing an entire cell. **b**, **c** 100k× magnification of the cell focusing on exosomes surrounding the cell. Black arrows point to exosomes. **d** Red fluorescently labeled exosomes concentrate to the green EYFP fibroblast cells and over a period of 2.5 h, the red fluorescence starts to diminish. **e** Control green EYFP cells tracked over time without any exosomes. **f** Magnified images at 30 min, 90 min (1.5 h), and 180 min (3 h) displaying a gradual decrease in red fluorescence and a complete lack of red fluorescence by 3 h. *N* = 3

As illustrated by the results of our in-vitro experiments, whole exosome treatment of fibroblasts leads to improved cell migration and cell viability; hence, we next investigated how exosomes interact with the cells and their mechanism of action. We performed TEM where exosomes were labeled with CD81 antibody and then treated with immunogold nanoparticles. Our TEM images illustrate that exosomes surround the fibroblast cell surface (Fig. 2a) and adhere to the surface in its intact form within 30 min (Fig. 2b, c). To determine the fate of the exosomes once they have adhered to the cell surface, we fluorescently labeled exosomal RNA with a red fluorophore and treated onto green fluorescent EYFP cells. We tracked the cells over time to monitor exosomes and cell interaction compared with EYFP cells without the treatment with exosomes. Results show a decrease in red fluorescent exosomes over time in the exosome treated group (Fig. 2d, e). Our time-lapse imaging shows that exosomes concentrate to the cell periphery and, by 3 h, the exosomes are mostly internalized by the cells as shown by a decrease in red fluorescence (Fig. 2f, Additional file 3: Video S1).

### AGWJ-derived exosomes enhance wound healing in vivo

**Fig. 3:**
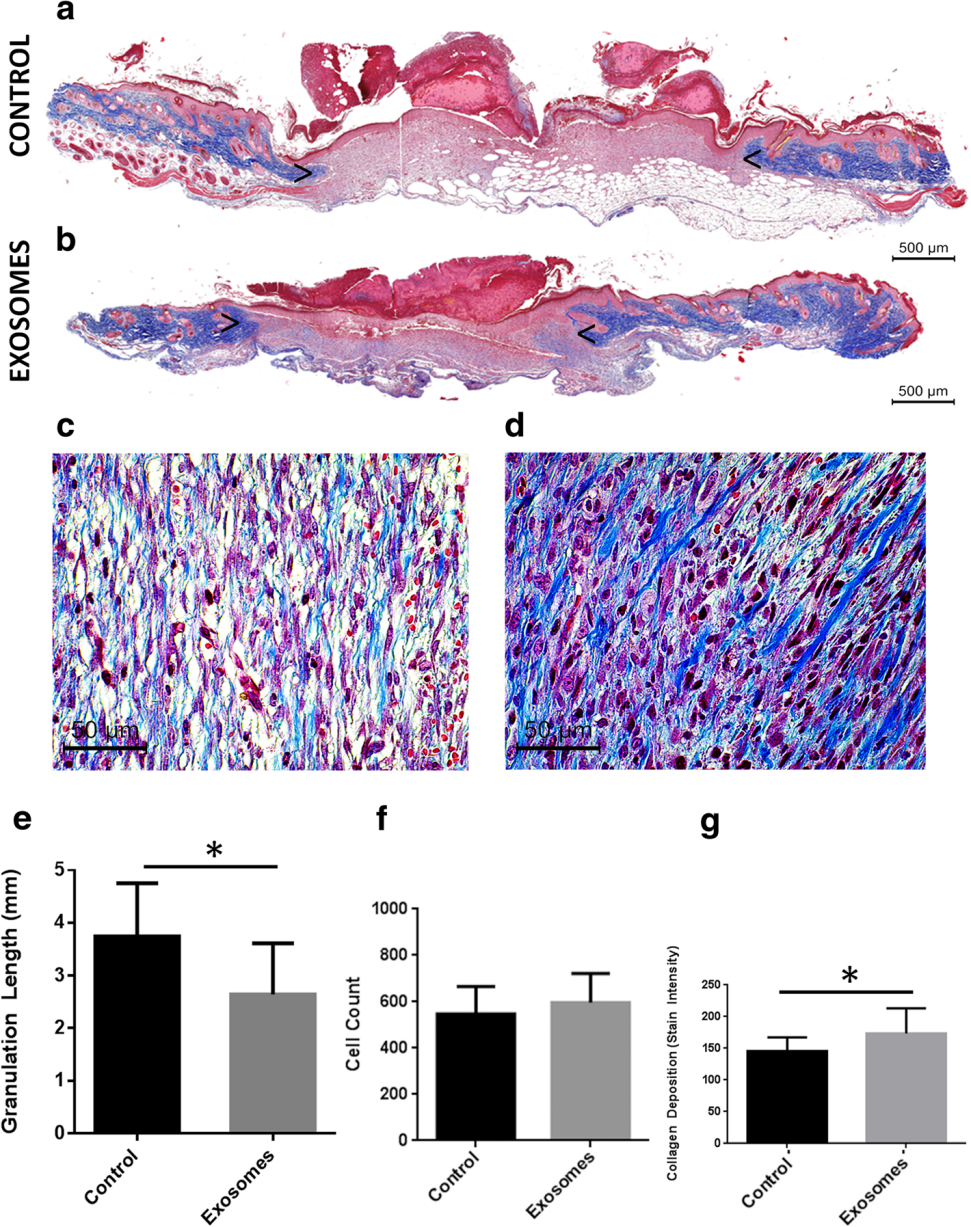
Effect of exosome treatment on wound healing in vivo after 7 days. **a** A representative image of a Masson’s trichrome stained wound displaying the length of a control wound. Complete wound images were scanned under 2.5× magnification. **b** A representative exosome-treated wound displaying the healing progress at the end of 7 days. **c** Representative 40× image from the center of the granulation tissue of a control wound displaying cells and granulation within the wound center. **d** Representative 40× image from the center of the granulation tissue of exosome-treated wound, displaying cells and granulation within the wound center. **e** Quantification of the measurement of the granulation length from blue to blue across the wound. **f** Quantification of the cell number within the wound bed from 40× images. **g** Quantification of blue granulation intensity from within the wound center, determined by a score out of 10 by three blinded individuals, 1 being lowest and 10 being highest. *N* = 4 control mice, *N* = 4 exosome-treated mice. Arrow heads point to the start and end of granulation tissue within the wound bed. * *p* < 0.05

Following the elucidation of exosome intercations with fibroblasts in vitro, we conducted a full-thickness skin biopsy experiment on the murine model. The wounds on the backs of the mice were treated with either control matrigel or matrigel containing exosomes. Trichrome staining of the excised wounds measuring the wound length and the granulation tissue demonstrates that exosome treatment significantly enhanced wound healing compared with the control treatment (Fig. 3a, b, e). The cell count inside the wound bed determined that there is a greater number of cells in the wound bed of the exosome-treated wounds compared with the control treatment. However, this number was not significantly higher when compared with the control treatment (Fig. 3f). Collagen deposition quantification revealed that exosome-treated wounds show a significantly higher collagen deposition compared with the control treatment (Fig. 3c, d, g).

### Exosome secretome contains proteins that are associated with wound healing

**Fig. 4:**
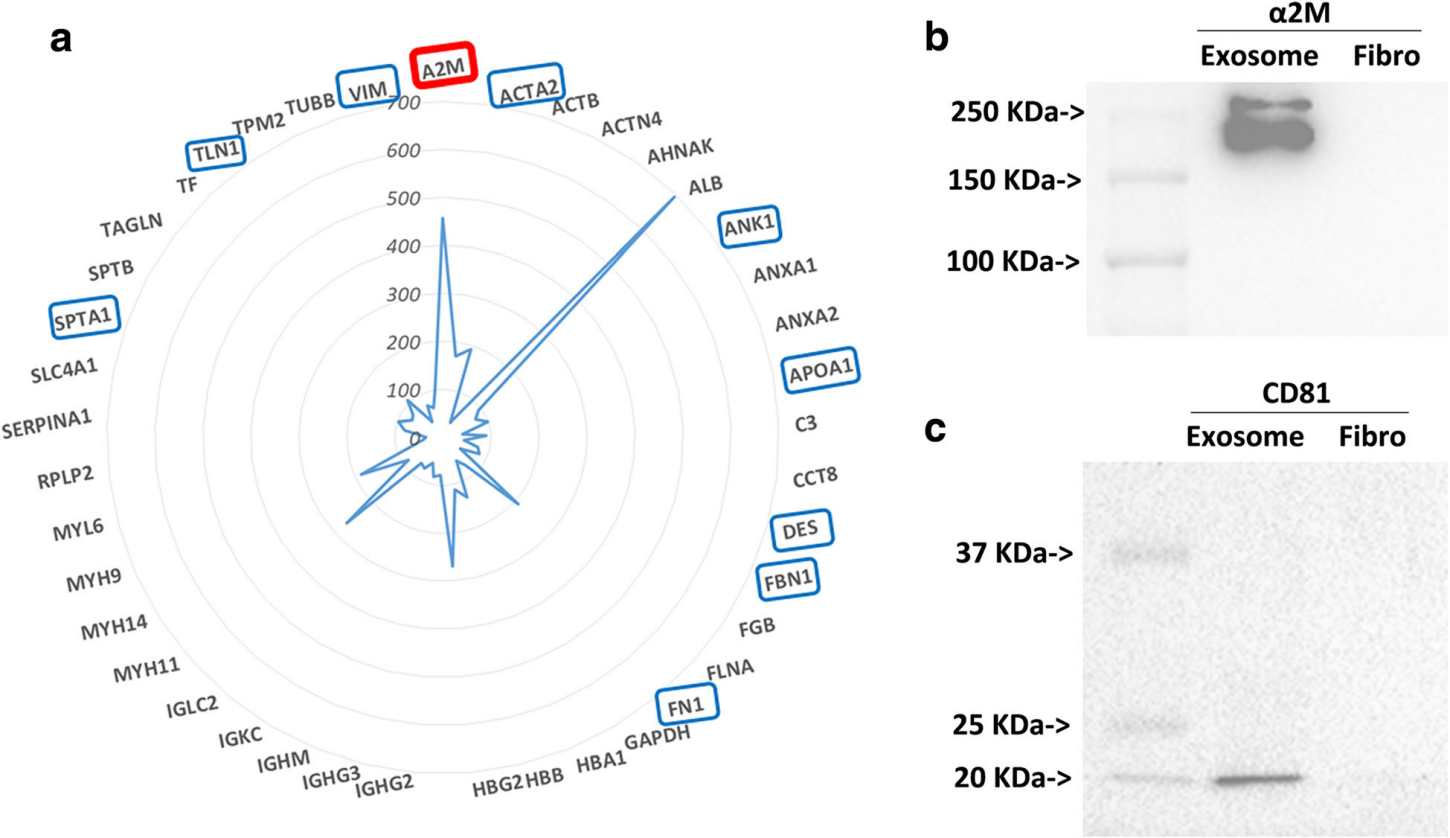
Proteomic analysis of exosomes. **a** Mass spectrometry analysis of exosomal proteins. Central star points to the two most abundant proteins, albumin (ALB) and alpha-2-macroglobulin (α2M). Blue boxes identify proteins involved in wound healing. **b** Western blot analysis confirming the expression of α2M in exosomes compared with fibroblast (Fibro) cells. **c** Western blot analysis confirming the presence of exosomes by detecting CD81 protein, a marker of exosomes. *N* = 3

In an effort to verify which proteins are contained in these acellular Wharton’s jelly-derived exosomes, we performed proteomic analysis through mass spectrometry on exosomes. The mass spectrometry analysis resulted in an extensive list of proteins, of which we investigated proteins involved in wound healing (Fig. 4a). Proteins such as actin aortic smooth muscle, vimentin, ankyrin, fibrillin, fibronectin, myosin 14, and desmin were present. Interestingly, the protein α2M had the highest yield in our samples after serum albumin (Alb), and therefore we investigated α2M in the context of wound healing. Western blot analysis on exosomes from umbilical cord AGWJ and fibroblasts isolated from normal skin confirmed that exosomes have a significant level of α2M protein compared with the lysate from fibroblasts (Fig. 4b). The exosomes were positive for CD81 protein, and the fibroblasts were negative for CD81 protein, further corroborating our results that the samples are indeed exosomes as compared to fibroblast cells (Fig. 4c).

### Alpha-2-macroglobulin enhances cell proliferation, migration, and cell viability in vitro

**Fig. 5:**
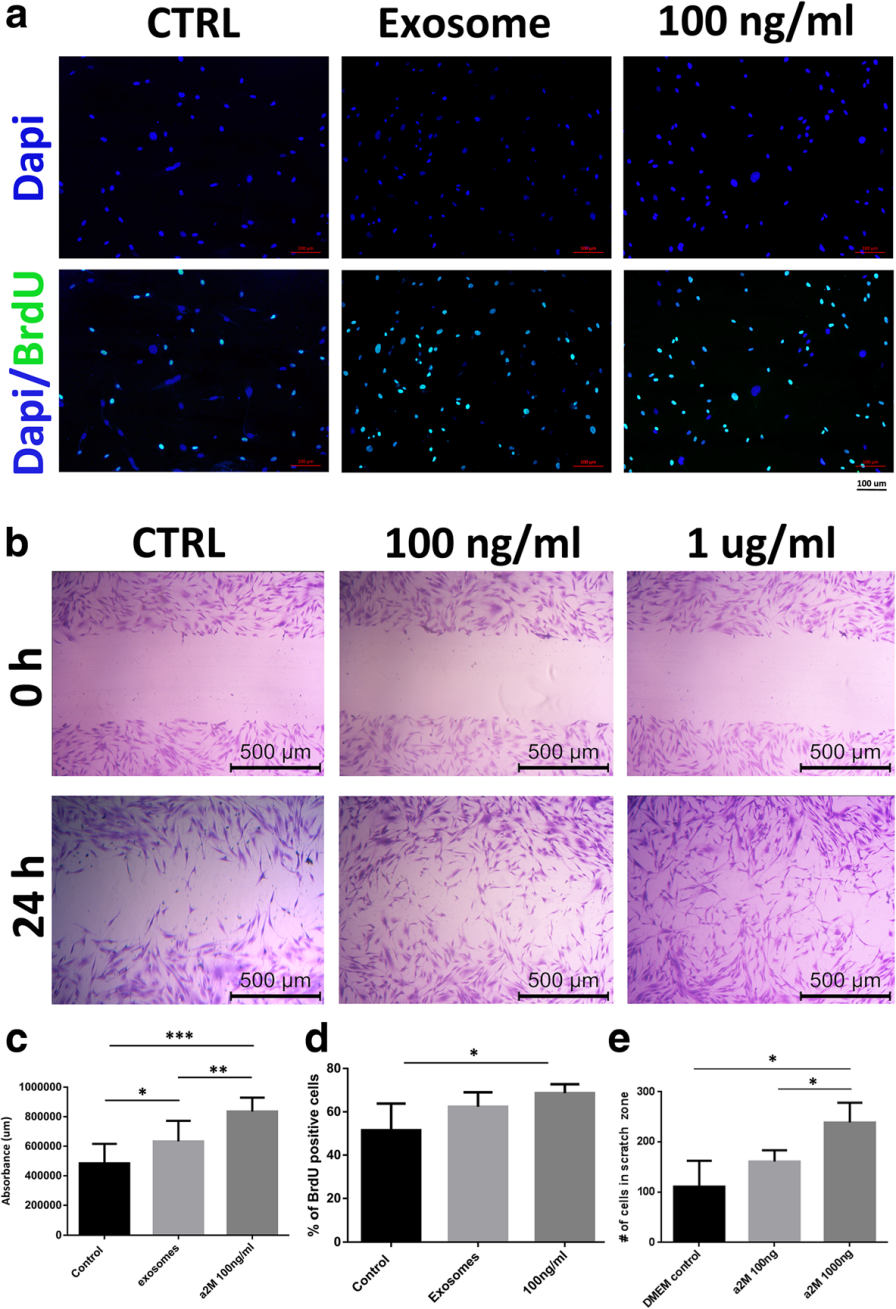
Effect of α2M on cell proliferation, migration, and viability. **a** Immunofluorescence imaging of bromodeoxyuridine (BrdU) incorporation into proliferating cells. DAPI labels the nucleus blue. The row labeled DAPI/BrdU displays the merged images after green FITC-labeled BrdU labels the nucleus of proliferating cells green. The treatments (labeled at the top) were either control (CTRL) DMEM-treated fibroblast cells, exosome-treated cells, or recombinant α2M-treated cells at 100 ng/ml. Images captured under 10× magnification. **b** Fibroblast scratch assay. Top row images were taken at 0 h and bottom row images were taken at 24 h time points. Treatments were either control (CTRL) DMEM-treated cells, α2M treatment at 100 ng/ml, and α2M treatment at 1 μg/ml. Images captured at 4× magnification. **c** Quantification of fibroblast cell viability. **d** Quantification of the percentage of BrdU-positive cells. **e** Quantification of the number of cells within the scratch zone. *N* = 4; *p < 0.05, **p < 0.01, ***p < 0.001

Considering the abundance of α2M in exosomes, we examined whether exogenously adding α2M to cells in vitro has any effect on their behavior with regards to wound healing characteristics. We established whether cell proliferation was affected by the treatment of α2M. Treatment of fibroblasts with control DMEM, exosomes, or a concentration of 100 ng/ml α2M for 24 h revealed that 100 ng/ml α2M significantly enhance BrdU incorporation into fibroblasts when compared with control (Fig. 5a, d). No significant difference was observed between exosome-treated cells in comparison with α2M-treated cells.

We next analyzed cell migration ability by conducting a scratch assay. Our results showed that, unlike cell proliferation that was enhanced with 100 ng/ml α2M, cell migration did not become enhanced with the low dose of α2M (Fig. 5b, e). However, adding α2M at the high dosage of 1000 ng/ml (1 μg/ml) enhanced cell migration significantly compared with control cells treated with complete DMEM (Fig. 5b).

The final outcome of this treatment, viability, was studied by treating cells with α2M. For this viability experiment, we treated both fibroblasts and keratinocytes with control media, exosomes, and 100 ng/ml and 1000 ng/ml α2M. The results illustrated that treatment of fibroblasts (Fig. 5c) and keratinocytes (Additional file 4: Figure S3) with 100 ng/ml α2M significantly enhanced cell viability.

## Discussion

We investigated the contents of acellular gelatinous Wharton’s jelly (AGWJ) of the human umbilical cord and its effect on skin wound healing. For this study, we conducted proteomic analysis on AGWJ by running mass spectrometry on umbilical cord AGWJ samples to establish which proteins are present in AGWJ and in what quantities. Our results revealed that isoform 2 of filamin-A was the most abundantly present in all samples, a protein expressed in extracellular exosomes. We hypothesized that exosomes are present in the AGWJ and these exosomes contain valuable cargo which is helping with the wound healing. We, therefore, proceeded to isolate the exosomes from the AGWJ.

We previously reported that AGWJ of the human umbilical cord enhances wound healing in vitro and in vivo in mice. We showed that the AGWJ material enhances cell migration in vitro and changes the morphology of the cells to a more myofibroblastic phenotype confirmed by the upregulation of αSMA. In vivo, AGWJ treatment of wounds led to a smaller wound length and an accelerated differentiation of fibroblasts to myofibroblasts in the wound, which led to a shorter proliferation phase [43].

Since our mass spectrometry results revealed that exosomal proteins were present in AGWJ, we hypothesized that exosomes exist in AGWJ. For the first time, we report on a new source for isolation of exosomes—the acellular Wharton’s jelly from within the umbilical cord. Although we cannot comment on the cellular source of these exosomes, it is likely that the exosomes originate from the WJ-MSCs since the AGWJ is the niche of WJ-MSCs. It has been reported that, in vitro, MSC-conditioned medium contains exosomes and these exosomes have beneficial therapeutic properties [41, 42]. However, we isolated exosomes directly from the native source and not from in-vitro culture. Exosomes are receiving a lot of attention because of their paracrine signaling properties and novel mode of noncellular therapy [39]. It has been shown that human umbilical cord MSC (hucMSC)-secreted exosomes (hucMSC-Ex) protect against liver fibrosis in vivo and also alter epithelial to mesenchymal transition (EMT) markers by increasing E-cadherin-positive cells. N-cadherin- and vimentin-positive cells decreased after hucMSC-Ex transplantation in mice [46]. However, in this previous study, the exosomes were derived from hucMSCs in culture and, unlike our study, they were not harvested from their native source [46]. Furthermore, hucMSC-Ex have been shown to have a beneficial effect on skin wound healing. It was reported that hucMSC-Ex-treated wounds dramatically increased re-epithelialization, with elevated expression of CK19, PCNA, and collagen I in vivo in a rat second-degree burn model [47]. HucMSC-Ex promoted the proliferation of skin cells and inhibited apoptosis of cells after heat stress in vitro [47]. Moreover, Wnt4 was established to be housed in hucMSC-Ex, and hucMSC-Ex-derived Wnt4 promoted β-catenin nuclear translocation and activity to increase proliferation and migration of skin cells [47], a critical pathway that is activated during skin healing [5, 13, 14, 48, 49]. Beside the mesenchymal milieu, in the hematopoietic niche Hu et al. showed that umbilical cord blood plasma exosomes (UCB-Exo) transplantation into mouse skin wounds leads to enhanced re-epithelialization, smaller scar widths, and greater angiogenesis [50]. It is yet to be clarified whether these observations are a secondary consequence of enhanced granulation tissue formation or if there is a direct effect on re-epithelialization and/or angiogenesis. In vitro, UCB-Exo increased fibroblast proliferation and migration and enhanced the angiogenic activities of endothelial cells. Quantitative real-time polymerase chain reaction analysis detected that miR-21-3p was highly enriched in UCB-Exo and served as a critical mediator in the regulatory effects of UCB-Exo [50]. Despite the fact that recent reports are highlighting the positive effect of exosomes from umbilical cord during skin healing, none of them use the native exosome isolated from tissue.

We postulated that exosomes were functional either in their whole intact form, or they were lysed open and then their contents exerted the effect of the exosomes. A cell viability assay and a cell migration assay were carried out using fibroblasts treated with either whole intact exosomes or fibroblasts treated with lysed exosomes. Interestingly, our results revealed that cell viability and migration were significantly improved when whole exosomes were added to the cells; however, the effect was diminished when the exosomes were lysed open. This suggested that the exosomes have to remain intact, perhaps because of the exosomal membrane to cell membrane interaction or because the exosome contents need to be delivered in a concentrated form which cannot happen if the exosomes are lysed open. We then conducted an in-vivo study on mice to determine if the results we observed in vitro can be translated to an in-vivo model. We added whole exosomes to full-thickness punch biopsy wounds in the mice as our treatment group, and added DMEM in matrigel as the control group. The results were in line with our in-vitro data. The wounds on the mice treated with exosomes established a shorter wound length compared with control wounds, likely due to enhanced healing. The granulation tissue also had more collagen deposition in the exosome-treated wounds compared with the controls, providing more evidence that exosomes enhanced wound healing. Zhang et al. also demonstrated that human-induced pluripotent stem cell-derived MSC exosomes enhanced collagen synthesis in the rat wound model [51]. They further showed in vitro that the exosomes increase fibroblast proliferation in a dose-dependent manner [51].

To further investigate the mode of exosome action, we visualized how the exosomes were interacting with cells with TEM analysis and then, through time-lapse imaging, we determined that the exosomes adhere and concentrate to the cell surface and, after 1 h, the exosomes were slowly being taken up into the cells. By 3 h, almost all of the exosomes were internalized by the cells, determined through a decrease and complete elimination of the red fluorescence used to mark the exosomes. Zhao et al. showed that human amniotic epithelial cell exosomes were internalized by fibroblasts and appeared in the perinuclear region, visualized with the PKH26 red fluorescent dye [52]. However, unlike our study, Zhao et al. did not determine the time course of exosomal interaction with fibroblasts or the timing of their eventual uptake into the cells. Their results do support our data that exosomes from the umbilical cord have wound healing benefits in the rat [52]. Another study by Wang et al. showed that exosomes secreted by human adipose mesenchymal stem cells cause extracellular matrix reconstruction by altering the ratios of collagen type III to type I, transforming growth factor (TGF)-β3 to TGF-β1, and MMP3 to TIMP1, and by regulating fibroblast differentiation into myofibroblasts to reduce scar formation [53]. These studies support our aim of establishing exosomes as a beacon of therapeutic potential for wound healing.

We identified key components within the exosomes through a proteomics approach—more specifically, mass spectrometry analysis of exosome samples. Mass spectrometry results identified an array of proteins expressed in the exosomes, some of which were involved in wound healing such as: actin aortic smooth muscle, vimentin, ankyrin, fibrillin, fibronectin, myosin 14, and desmin; however, α2M was a protein that was highly abundant among all of our exosome samples. While all the aforementioned proteins might affect wound healing, little is known about the role of α2M. α2M is a tetrameric plasma glycoprotein that inhibits four classes of proteinases by a ‘trapping’ mechanism [54, 55].

During skin healing, there is a dynamic interplay between cells in granulation tissue and extracellular matrices. While dermal fibroblasts contribute to the secretion of extracellular matrix components [56], there is a feedback loop where components of extracellular matrices affect fibroblasts and activate some of the signaling pathways important for wound healing such as wnt/β-catenin signaling [57]. The abundance of α2M in exosomes, together with the promising effect of exosomes on wound healing in vitro and in vivo, raises the possibility that exosomes enhance wound healing, at least partly, through the paracrine effects of α2M. We exogenously added α2M to cells after conducting a scratch assay to determine its effect on cell migration. Using different doses of α2M [58] it was revealed that the high dose of 1000 ng/ml had the greatest effect on cell migration into the scratch zone while the lower doses did not affect cell migration. It is likely that exosomes containing an abundant amount of α2M in their cargo are engulfed by cells in the wound area which enhances cell migration. Our viability assay with 100 ng/ml α2M exogenously added to fibroblasts showed that α2M had the greatest increase in viability compared with whole exosomes and controls. For keratinocytes, similarly, 100 ng/ml showed significant improvement in keratinocyte viability. These data suggest that α2M increases two important characteristics of cells during healing (migration and viability) and this might be the underlying mechanism behind the positive effect of exosomes on wound healing that we herein report. It is not clear whether the inhibition of proteinases, and hence an increase in growth factors, is affecting the cellular characteristics in favor of wound healing or if α2M directly affects the cells.

## Conclusions

This study identifies exosomes as being enriched in the native niche of the umbilical cord, the AGWJ. For the first time, we have harvested exosomes from its biological niche within the umbilical cord AGWJ and established that the exosomes play a critical role in aiding wound healing in vitro and in vivo. We went further and established the exosomal mechanism of action by tracking exosomal interaction with cells in real time. We then delved deeper and identified the abundant proteins within the exosome cargo using a proteomics approach and discovered that alpha-2-macroglobulin was highly present in all of the AGWJ-derived exosomes that we analyzed. Adding exogenous α2M protein to cells revealed that α2M might aid in wound healing by enhancing cell migration, viability, and proliferation, the key properties of skin wound healing. Exosomes from the AGWJ and their cargo protein α2M have tremendous potential as being a noncellular off-the-shelf therapeutic modality for wound healing.

## Additional files

Additional file 1: **Figure S1.** Scanning electron microscopy (SEM) images of cord cross-section. SEM images show two zones. Zone 1 displays a section in between the umbilical vein and two arteries, and zone 2 displays a region closer to the umbilical vein. (A–E) Magnification of zone 1. (F–J) Magnification of zone 2. (TIF 10880 kb)

Additional file 2: **Figure S2.** Schematic representation of the exosome isolation protocol. (TIF 3668 kb)

Additional file 3: **Video S1.** Time-lapse imaging showing that exosomes concentrate to the cell periphery and, by 3 h, disappear as depicted by a decrease in red fluorescence. (WMV 32791 kb)

Additional file 4: **Figure S3.** Cell viability quantification for keratinocytes. Control medium-treated keratinocytes compared with exosome-treated and α2M (100 ng/ml)-treated keratinocytes. *p < 0.05, ***p < 0.001. *N* = 6 for control and exosomes; *N* = 12 for α2M. (TIF 4635 kb)

## Abbreviations

AGWJ: Acellular gelatinous Wharton’s jelly
BrdU: Bromodeoxyuridine
DMEM: Dulbecco’s modified Eagle’s medium
EMT: Epithelial to mesenchymal transition
FBS: Fetal bovine serum
HSC: Hematopoietic stem cell
hucMSC: Human umbilical cord mesenchymal stromal cell
huc-MSC-Ex: Human umbilical cord mesenchymal stromal cell-secreted exosomes
MS: Mass spectrometry
MSC: Mesenchymal stromal cell
PBS: Phosphate-buffered saline
SEM: Scanning electron microscopy
SMA: Smooth muscle actin
TEM: Transmission electron microscopy
UCB-Exo: Umbilical cord blood plasma exosomes
WJ: Wharton’s jellyα2M Alpha-2-macroglobulin

## References

[1] Bielefeld KA, Amini-Nik S, Alman BA (2013). Cutaneous wound healing: recruiting developmental pathways for regeneration. Cellular and molecular life sciences : CMLS.

[2] Singer AJ, Clark RAF (1999). Cutaneous wound healing. N Engl J Med.

[3] W.H.O. (WHO), in, http://www.who.int/en/news-room/fact-sheets/detail/burns, 2018.

[4] Sadiq A, Shah A, Jeschke MG, Belo C, Qasim Hayat M, Murad S, Amini-Nik S. The role of serotonin during skin healing in post-thermal injury. Int J Mol Sci. 2018;19 10.3390/ijms19041034 PMC5979562 29596386

[5] Jeschke MG, Patsouris D, Stanojcic M, Abdullahi A, Rehou S, Pinto R, Chen P, Burnett M, Amini-Nik S (2015). Pathophysiologic response to burns in the elderly. EBioMedicine.

[6] Jeschke MG, Pinto R, Costford SR, Amini-Nik S (2016). Threshold age and burn size associated with poor outcomes in the elderly after burn injury. Burns : journal of the International Society for Burn Injuries.

[7] Valacchi G, Zanardi I, Sticozzi C, Bocci V, Travagli V (2012). Emerging topics in cutaneous wound repair. Ann N Y Acad Sci.

[8] Fahey TJ, Sadaty A, Jones WG, Barber A, Smoller B, Shires GT (1991). Diabetes impairs the late inflammatory response to wound healing. J Surg Res.

[9] Singer AJ, Clark RA (1999). Cutaneous wound healing. N Engl J Med.

[10] Shah A, Alhusayen R, Amini-Nik S. The critical role of macrophages in the pathogenesis of hidradenitis suppurativa. Inflammation research : official journal of the European Histamine Research Society [et al]. 2017; 10.1007/s00011-017-1074-y 28656364

[11] Shah A, Amini-Nik S (2017). The role of serotoninergic system in skin healing. International Journal of Drug Research and Technology.

[12] Gosain A, DiPietro LA (2004). Aging and wound healing. World J Surg.

[13] Amini-Nik S, Cambridge E, Yu W, Guo A, Whetstone H, Nadesan P, Poon R, Hinz B, Alman BA (2014). Beta-catenin-regulated myeloid cell adhesion and migration determine wound healing. J Clin Invest.

[14] Amini-Nik S, Yousuf Y, Jeschke MG (2018). Scar management in burn injuries using drug delivery and molecular signaling: current treatments and future directions. Adv Drug Deliv Rev.

[15] Tredget EE, Nedelec B, Scott PG, Ghahary A (1997). Hypertrophic scars, keloids, and contractures. The cellular and molecular basis for therapy. Surg Clin North Am.

[16] Nicholas MN, Jeschke MG, Amini-Nik S (2016). Methodologies in creating skin substitutes. Cellular and molecular life sciences : CMLS.

[17] Sheikholeslam M, Wright MEE, Jeschke MG, Amini-Nik S. Biomaterials for skin substitutes. Advanced healthcare materials. 2017; 10.1002/adhm.201700897 PMC7863571 29271580

[18] Nicholas MN, Jeschke MG, Amini-Nik S (2016). Cellularized bilayer pullulan-gelatin hydrogel for skin regeneration. Tissue Eng A.

[19] Hakimi N, Cheng R, Leng L, Sotoudehfar M, Ba PQ, Bakhtyar N, Amini-Nik S, Jeschke MG, Gunther A (2018). Handheld skin printer: in situ formation of planar biomaterials and tissues. Lab Chip.

[20] Arno AI, Amini-Nik S, Blit PH, Al-Shehab M, Belo C, Herer E, Jeschke MG (2014). Effect of human Wharton's jelly mesenchymal stem cell paracrine signaling on keloid fibroblasts. Stem Cells Transl Med.

[21] Arno AI, Amini-Nik S, Blit PH, Al-Shehab M, Belo C, Herer E, Tien CH, Jeschke MG (2014). Human Wharton's jelly mesenchymal stem cells promote skin wound healing through paracrine signaling. Stem Cell Res Ther.

[22] Meyer FA, Laver-Rudich Z, Tanenbaum R (1983). Evidence for a mechanical coupling of glycoprotein microfibrils with collagen fibrils in Wharton's jelly. Biochim Biophys Acta.

[23] Sakamoto T, Ono H, Saito Y (1996). Electron microscopic histochemical studies on the localization of hyaluronic acid in Wharton's jelly of the human umbilical cord. Nihon Sanka Fujinka Gakkai zasshi.

[24] Sobolewski K, Bankowski E, Chyczewski L, Jaworski S (1997). Collagen and glycosaminoglycans of Wharton's jelly. Biol Neonate.

[25] Troyer DL, Weiss ML (2008). Wharton's jelly-derived cells are a primitive stromal cell population. Stem Cells.

[26] McElreavey KD, Irvine AI, Ennis KT, McLean WH. Isolation, culture and characterisation of fibroblast-like cells derived from the Wharton's jelly portion of human umbilical cord. Biochem Soc Trans. 1991;19, 29s 10.1042/bst019029s 1709890

[27] Pirjali T, Azarpira N, Ayatollahi M, Aghdaie MH, Geramizadeh B, Talai T (2013). Isolation and characterization of human mesenchymal stem cells derived from human umbilical cord Wharton's jelly and amniotic membrane. Int J Organ Transplant Med.

[28] H.S. Wang, S.C. Hung, S.T. Peng, C.C. Huang, H.M. Wei, Y.J. Guo, Y.S. Fu, M.C. Lai, C.C. Chen, Mesenchymal stem cells in the Wharton's jelly of the human umbilical cord, Stem Cells, 22 (2004) 1330–1337. 10.1634/stemcells.2004-0013 15579650

[29] Sabapathy V, Sundaram B, Mankuzhy P, Kumar S, M.S. V (2014). Human Wharton's jelly mesenchymal stem cells plasticity augments scar-free skin wound healing with hair growth. PLoS One.

[30] da Silva CL, Goncalves R, Crapnell KB, Cabral JM, Zanjani ED, Almeida-Porada G (2005). A human stromal-based serum-free culture system supports the ex vivo expansion/maintenance of bone marrow and cord blood hematopoietic stem/progenitor cells. Exp Hematol.

[31] Ito Y, Hasauda H, Kitajima T, Kiyono T (2006). Ex vivo expansion of human cord blood hematopoietic progenitor cells using glutaraldehyde-fixed human bone marrow stromal cells. J Biosci Bioeng.

[32] Raposo G, Stoorvogel W (2013). Extracellular vesicles: exosomes, microvesicles, and friends. J Cell Biol.

[33] Venkat P, Chopp M, Chen J. Cell-based and exosome therapy in diabetic stroke. Stem Cells Transl Med. 2018; 10.1002/sctm.18-0014 PMC5980126 29498242

[34] Thery C, Zitvogel L, Amigorena S (2002). Exosomes: composition, biogenesis and function. Nat Rev Immunol.

[35] Admyre C, Johansson SM, Qazi KR, Filen JJ, Lahesmaa R, Norman M, Neve EP, Scheynius A, Gabrielsson S (2007). Exosomes with immune modulatory features are present in human breast milk. Journal of immunology (Baltimore, Md : 1950).

[36] Caby MP, Lankar D, Vincendeau-Scherrer C, Raposo G, Bonnerot C (2005). Exosomal-like vesicles are present in human blood plasma. Int Immunol.

[37] Park KH, Kim BJ, Kang J, Nam TS, Lim JM, Kim HT, Park JK, Kim YG, Chae SW, Kim UH (2011). Ca2+ signaling tools acquired from prostasomes are required for progesterone-induced sperm motility. Sci Signal.

[38] Ronquist G, Brody I (1985). The prostasome: its secretion and function in man. Biochim Biophys Acta.

[39] Bang C, Thum T (2012). Exosomes: new players in cell-cell communication. Int J Biochem Cell Biol.

[40] Simpson RJ, Jensen SS, Lim JW (2008). Proteomic profiling of exosomes: current perspectives. Proteomics.

[41] Lai RC, Arslan F, Lee MM, Sze NS, Choo A, Chen TS, Salto-Tellez M, Timmers L, Lee CN, El Oakley RM, Pasterkamp G, de Kleijn DP, Lim SK (2010). Exosome secreted by MSC reduces myocardial ischemia/reperfusion injury. Stem Cell Res.

[42] van Koppen A, Joles JA, van Balkom BW, Lim SK, de Kleijn D, Giles RH, Verhaar MC (2012). Human embryonic mesenchymal stem cell-derived conditioned medium rescues kidney function in rats with established chronic kidney disease. PLoS One.

[43] Bakhtyar N, Jeschke MG, Mainville L, Herer E, Amini-Nik S (2017). Acellular gelatinous material of human umbilical cord enhances wound healing: a candidate remedy for deficient wound healing. Front Physiol.

[44] Carayon K, Chaoui K, Ronzier E, Lazar I, Bertrand-Michel J, Roques V, Balor S, Terce F, Lopez A, Salome L, Joly E (2011). Proteolipidic composition of exosomes changes during reticulocyte maturation. J Biol Chem.

[45] Dang VD, Jella KK, Ragheb RRT, Denslow ND, Alli AA (2017). Lipidomic and proteomic analysis of exosomes from mouse cortical collecting duct cells. FASEB journal : official publication of the Federation of American Societies for Experimental Biology.

[46] Li T, Yan Y, Wang B, Qian H, Zhang X, Shen L, Wang M, Zhou Y, Zhu W, Li W, Xu W (2013). Exosomes derived from human umbilical cord mesenchymal stem cells alleviate liver fibrosis. Stem Cells Dev.

[47] Zhang B, Wang M, Gong A, Zhang X, Wu X, Zhu Y, Shi H, Wu L, Zhu W, Qian H, Xu W (2015). HucMSC-exosome mediated-Wnt4 signaling is required for cutaneous wound healing. Stem Cells.

[48] Amini-Nik S, Glancy D, Boimer C, Whetstone H, Keller C, Alman BA (2011). Pax7 expressing cells contribute to dermal wound repair, regulating scar size through a beta-catenin mediated process. Stem cells (Dayton, Ohio).

[49] Amini-Nik S, Kraemer D, Cowan ML, Gunaratne K, Nadesan P, Alman BA, Miller RJ. Ultrafast mid-IR laser scalpel: protein signals of the fundamental limits to minimally invasive surgery. PLoS One. 2010;5 10.1371/journal.pone.0013053 PMC2946918 20927391

[50] Hu Y, Rao SS, Wang ZX, Cao J, Tan YJ, Luo J, Li HM, Zhang WS, Chen CY, Xie H (2018). Exosomes from human umbilical cord blood accelerate cutaneous wound healing through miR-21-3p-mediated promotion of angiogenesis and fibroblast function. Theranostics.

[51] Zhang J, Guan J, Niu X, Hu G, Guo S, Li Q, Xie Z, Zhang C, Wang Y (2015). Exosomes released from human induced pluripotent stem cells-derived MSCs facilitate cutaneous wound healing by promoting collagen synthesis and angiogenesis. J Transl Med.

[52] Zhao B, Zhang Y, Han S, Zhang W, Zhou Q, Guan H, Liu J, Shi J, Su L, Hu D (2017). Exosomes derived from human amniotic epithelial cells accelerate wound healing and inhibit scar formation. J Mol Histol.

[53] Wang L, Hu L, Zhou X, Xiong Z, Zhang C, Shehada HMA, Hu B, Song J, Chen L (2017). Exosomes secreted by human adipose mesenchymal stem cells promote scarless cutaneous repair by regulating extracellular matrix remodelling. Sci Rep.

[54] Sottrup-Jensen L, Stepanik TM, Kristensen T, Wierzbicki DM, Jones CM, Lonblad PB, Magnusson S, Petersen TE (1984). Primary structure of human alpha 2-macroglobulin. V. The complete structure. J Biol Chem.

[55] http://www.uniprot.org/uniprot/P01023, Uniprot, in, 2018.

[56] Clark RA (1990). Fibronectin matrix deposition and fibronectin receptor expression in healing and normal skin. The Journal of investigative dermatology.

[57] Bielefeld KA, Amini-Nik S, Whetstone H, Poon R, Youn A, Wang J, Alman BA (2011). Fibronectin and beta-catenin act in a regulatory loop in dermal fibroblasts to modulate cutaneous healing. J Biol Chem.

[58] Yi KW, Jung SH, Cho GJ, Seol HJ, Hong SC, Oh MJ, Kim HJ (2014). Effects of sFlt-1 and alpha 2-macroglobulin on vascular endothelial growth factor-induced endothelin-1 upregulation in human microvascular endothelial cells. Placenta.

