# Supplementary figures and images for: Exosomes from acellular Wharton’s jelly of the human umbilical cord promotes skin wound healing
Source: Stem Cell Res Ther. 2018 Jul 13;9:193. doi: 10.1186/s13287-018-0921-2 (PMC6044104; doi:10.1186/s13287-018-0921-2)

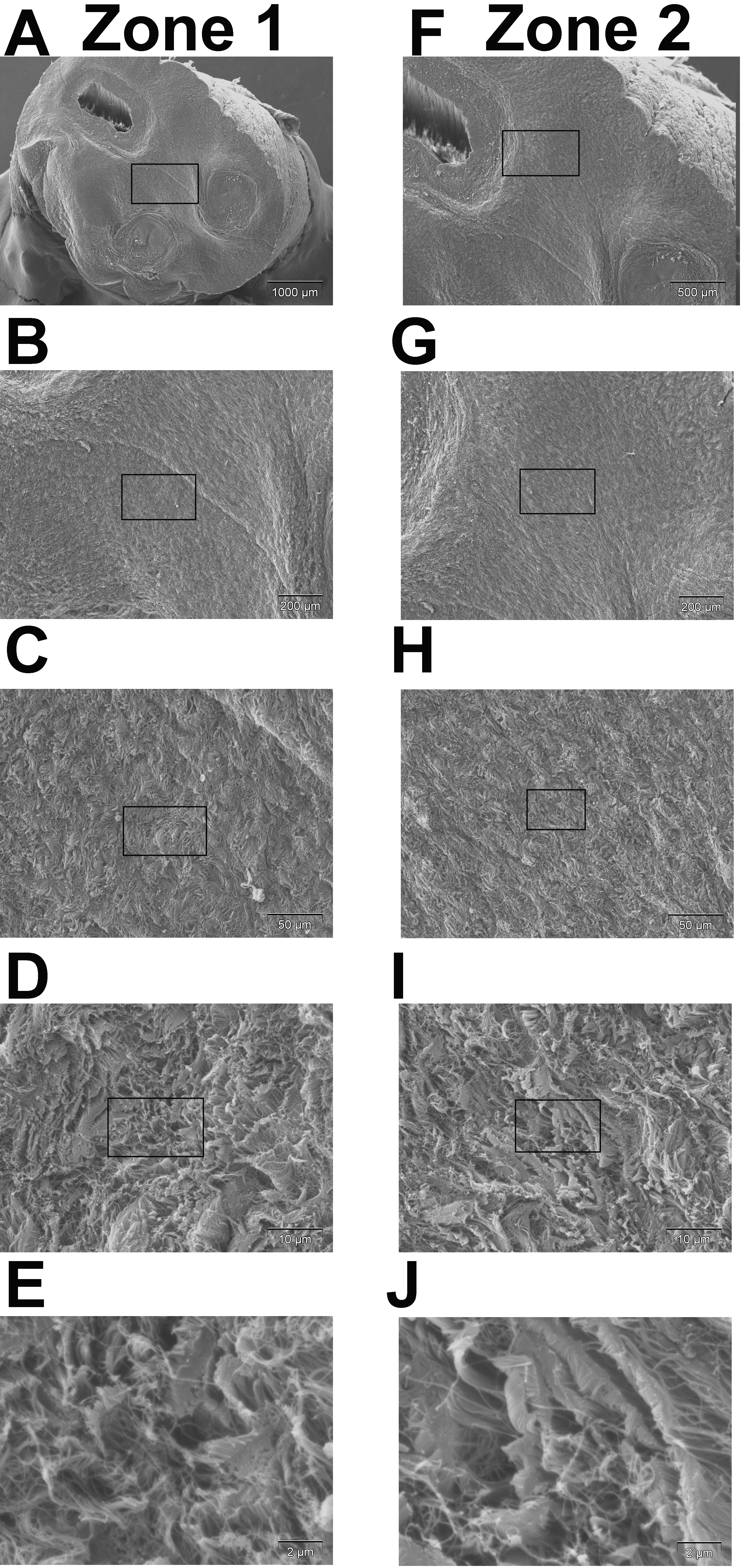

Supplement: Supplementary file 1 — Figure S1. Scanning electron microscopy (SEM) images of cord cross-section. SEM images show two zones. Zone 1 displays a section in between the umbilical vein and two arteries, and zone 2 displays a region closer to the umbilical vein. (A–E) Magnification of zone 1. (F–J) Magnification of zone 2. (TIF 10880 kb) [file 13287_2018_921_MOESM1_ESM.tif]

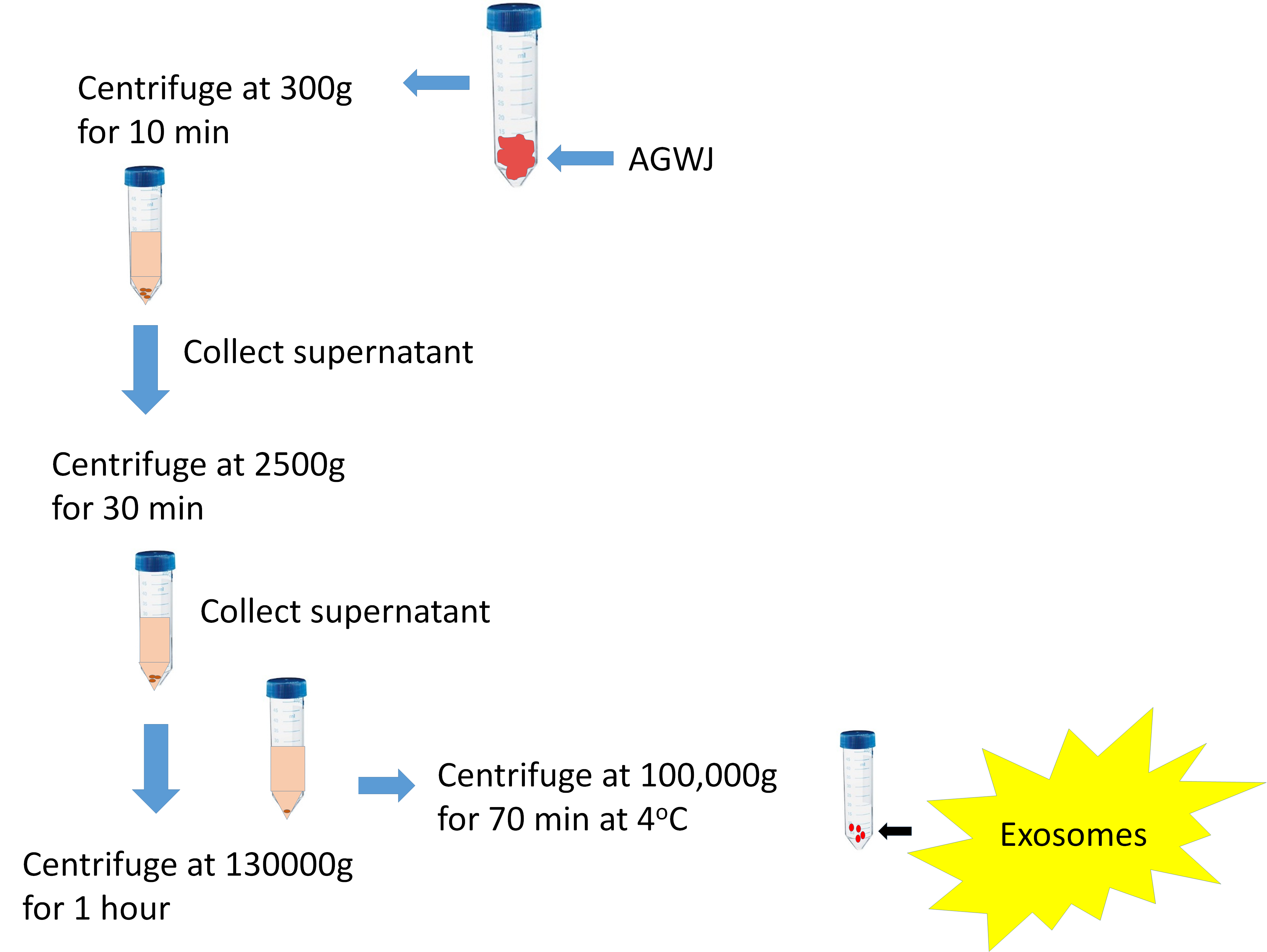

Supplement: Supplementary file 2 — Figure S2. Schematic representation of the exosome isolation protocol. (TIF 3668 kb) [file 13287_2018_921_MOESM2_ESM.tif]

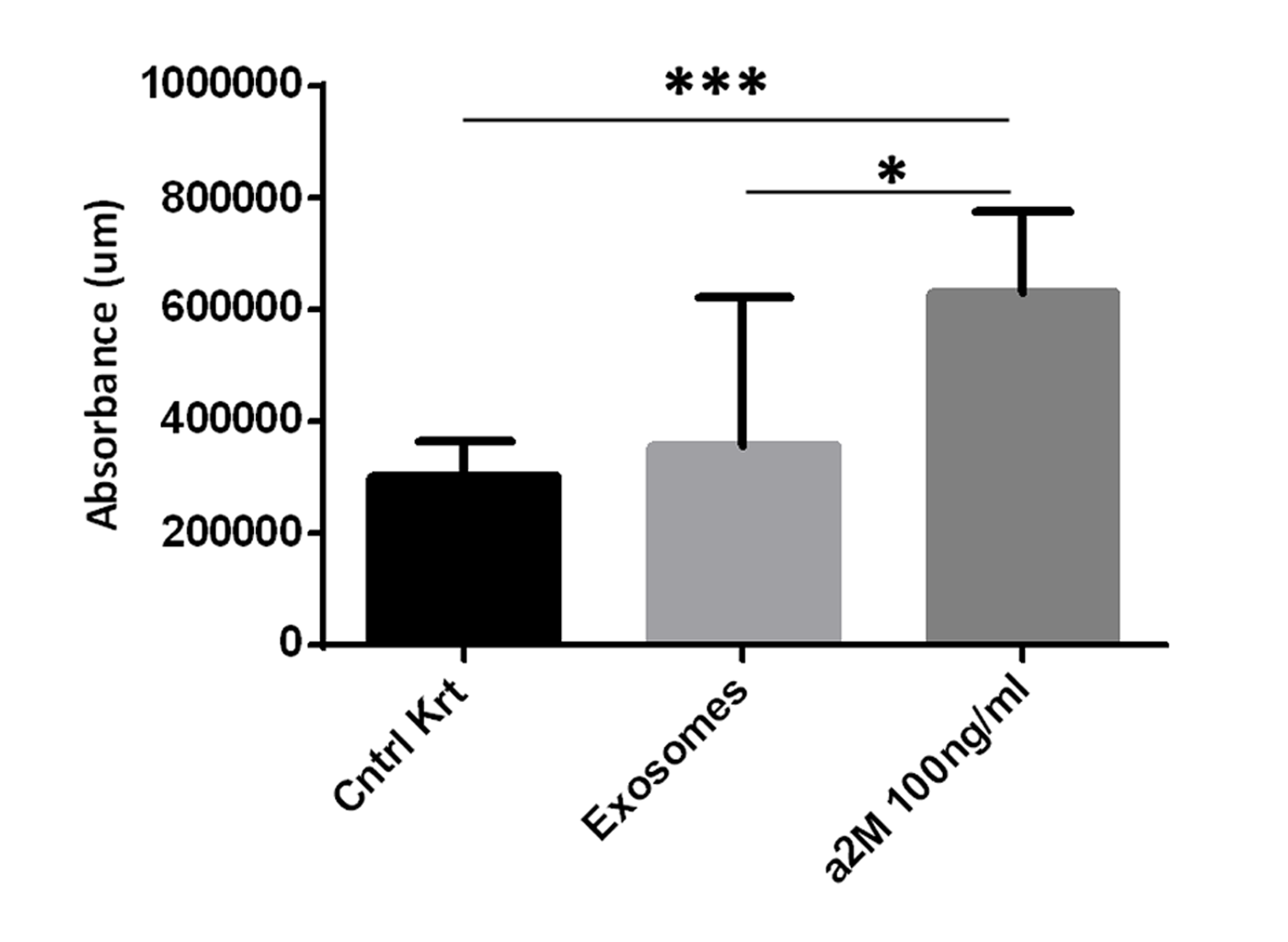

Supplement: Supplementary file 4 — Figure S3. Cell viability quantification for keratinocytes. Control medium-treated keratinocytes compared with exosome-treated and α2M (100 ng/ml)-treated keratinocytes. *p < 0.05, ***p < 0.001. N = 6 for control and exosomes; N = 12 for α2M. (TIF 4635 kb) [file 13287_2018_921_MOESM4_ESM.tif]
